# Pagets disease of uncertain origin: case report

**DOI:** 10.1186/1477-7800-4-12

**Published:** 2007-05-06

**Authors:** Ashok Subramanian, Hilary Birch, Rita McAvinchey, Adam Stacey-Clear

**Affiliations:** 1 Department of General Surgery, East Surrey Hospital, Canada Avenue, Redhill, Surrey, UK; 2 Department of Histopathology, East Surrey Hospital, Canada Avenue, Redhill, Surrey, UK; 3 Department of Radiology, East Surrey Hospital, Canada Avenue, Redhill, Surrey, UK

## Abstract

**Background:**

Pagets disease of the nipple presents as an eczematous lesion, occurs in 1 – 4% of all female breast carcinoma cases and is invariably associated with underlying malignancy either overt or occult. The majority of these cases are invasive disease although 40–45% are associated with DCIS.

**Case presentation:**

A 39 year old lady presented to our unit with a palpable lump in the right breast. Radiological and histological investigation proved this to be an extensive area of Ductal Carcinoma in Situ (DCIS) for which she underwent a simple mastectomy and immediate latissimus dorsi flap reconstruction. Histology revealed high grade DCIS with 2 small foci of invasive carcinoma. At 1 year the patient represented with a nodule adjacent to the reconstruction scar which was proved on biopsy to be consistent with Paget's disease. This was proved on formal excision.

**Conclusion:**

In the absence of underlying breast or apocrine tissue this case details a case of Paget's disease of uncertain origin.

## Background

Pagets disease of the nipple accounts for between 1–4% of all cases of female breast carcinoma and presents as a chronic eczematous change of the nipple often with an underlying palpable lump. Classically the underlying carcinoma is invasive in nature although in 40–45% of cases the underlying pathology is DCIS. A prerequisite to the development of this condition is the presence of the nipple or at least underlying mammary tissue both of which were absent in this case. We present a case of a 39 year old lady who re-presented with Pagets disease following mastectomy and latissimus dorsi reconstruction, in the absence of a nipple or underlying breast tissue.

## Case presentation

A 39 year old lady was referred to our breast unit with a one week history of a lump in the lateral aspect of the right breast. On examination, a generalized hardness was felt in the right breast together with central retraction of the nipple.

Mammography revealed malignant microcalcification affecting the whole breast with core biopsy proving high grade ductal carcinoma in situ (DCIS).

Following discussion at the multi disciplinary meeting, she underwent a mastectomy and axillary sampling together with an immediate latissimus dorsi (LD) reconstruction flap. Post-operative recovery was unremarkable and the cosmetic result was good.

Histology of the breast revealed 120 mm of high grade DCIS of mixed comedo and solid type. Two separate foci of invasive ductal carcinoma (Grade 1 and Grade 2) were identified. The margins were clear of tumour by 1.3 mm (in-situ disease) and 5.8 mm (invasive component). No evidence of Paget's disease was documented at this time. The axillary sample was negative for tumour.

**Figure 1:**
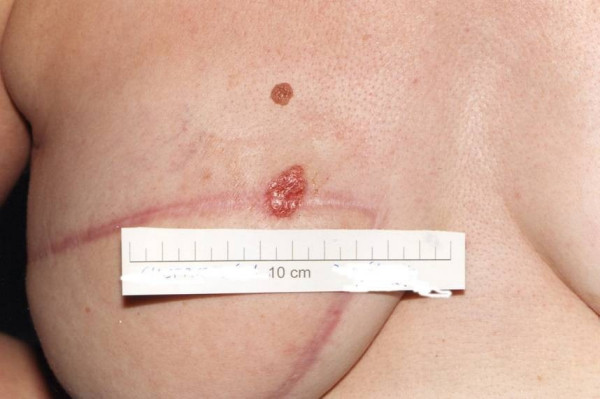
Recurrent nodule in the latissimus dorsi reconstruction scar.

As the tumour demonstrated strong Estrogen/Progesterone positivity, Mrs K.S was started on postoperative Tamoxifen treatment but this was discontinued within 6 months due to adverse side effects. On her second post-operative visit, a small erythematous skin lesion was noted on the superio-medial aspect of the flap adjacent to the scar (Fig 1).

A punch biopsy was obtained which revealed foci of atypical cells within the epidermis with Pagetoid spread. Immunostaining showed positivity for CAM 5.2, CEA and EMA, consistent with. Paget's disease.

**Figure 2:**
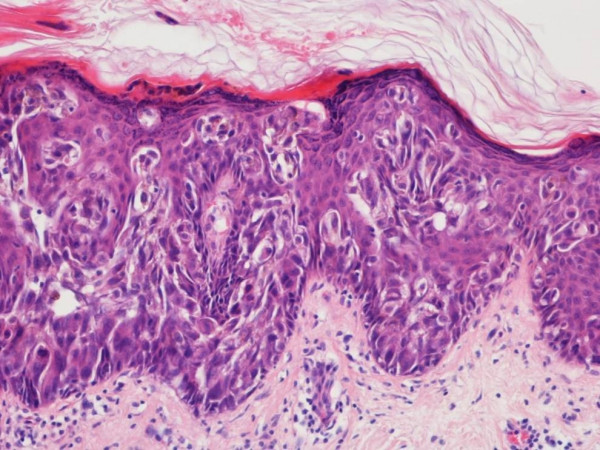
Histological section of skin showing malignant glandular epithelial cells within the epidermis (Paget's disease) H&E ×200.

A wide local excision of the lesion was performed and the specimen was sent for a histological second opinion. This showed acanthosis, hyperkeratosis with focal parakeratosis and extensive replacement of the epidermis by pleomorphic epithelial cells with marked pagetoid pattern (Fig 2). Tumour cells extended into adjacent hair follicles and immunohistochemistry confirmed features of Paget's disease.

The differential diagnosis was between that of Paget's disease of the breast (although there was no underlying breast tissue or nipple and it was not seen in the original mastectomy specimen) and extra-mammary Paget's disease of the skin.

Because of the past history of breast carcinoma, a diagnosis of Paget's disease of the breast was made.

## Conclusion

Paget's disease of the breast was first described by Sir James Paget in 1874 [1] who described "an eczematous change in the skin of the nipple preceeding an underlying mammary cancer". It occurs in 1 – 4% of all female breast carcinoma cases and is invariably associated with underlying malignancy either overt or occult. The majority of these cases are invasive disease although 40–45% are associated with DCIS [2].

Opinion is divided regarding the pathogenesis of this condition.

It is thought that malignant epithelial cells from intraductal carcinoma, extend into the overlying epidermis through mammary duct epithelium and proliferate in the epidermis causing thickening of the nipple and areolar skin. This is supported by the observation that Paget cells often share cell surface markers with the underlying breast carcinoma (e.g CAM 5.2, CEA, c-*erb *2 and EMA) [3,4]. Normal epidermal keratinocytes produce and release the mobility factor heregulin-alpha which is chemotactic for heregulin receptors (Her-2) and coreceptors Her 3 and Her 4 which are produced by Pagets cells. This is thought to result in migration of these cells to the nipple epidermis [5].

Others believe that Paget's cells are derived from clear nipple epithelium (Toker) cells [6] and that underlying intraductal carcinoma is simply coexisting with this disease [7].

Extramammary Paget's disease was first described by Radcliffe Crocker in 1889. It is histologically identical but anatomically different to mammary Paget's disease affecting sites rich in apocrine glands such as the genetalia, axillae, perineum, and external auditory canal [8,9]. Unlike mammary Paget's disease, the majority (75%) of cases arise de-novo as a primary cutaneous adenocarcinoma with the epidermis being infiltrated by neoplastic cells showing glandular differentiation. In the remaining 25% there is an associated underlying in-situ or invasive carcinoma, most commonly a primary adnexal apocrine carcinoma which produces the skin lesion as a secondary event [10].

In our case, following mastectomy and latissimus dorsi reconstruction, a focus of Paget's disease was found in the scar bridging the breast and transplanted skin. Neither of these areas are apocrine rich and therefore would be unlikely candidates for extramammary Paget's disease. In the absence of a a nipple, underlying ductal epithelium or breast tissue in general it is also very hard to explain as part of mammary Paget's disease.

In this case, neoplastic cells from an axillary apocrine carcinoma may have migrated up the hair follicle and along the surgical scar but the exact pathogenesis still remains unclear.
